# Poxvirus Vectors Activate Human NK and MAIT Cells in a Type I Interferon, IL-18, and Monocyte-Dependent Manner

**DOI:** 10.1155/jimr/1203141

**Published:** 2025-07-12

**Authors:** Kombo F. N'guessan, Zhanna Shubin, Kawthar Machmach, Johan K. Sandberg, Julie A. Ake, Sandhya Vasan, Michael A. Eller, Dominic Paquin-Proulx

**Affiliations:** ^1^US Military HIV Research Program, Center for Infectious Disease Research, Walter Reed Army Institute of Research, Silver Spring, Maryland, USA; ^2^Henry M. Jackson Foundation for the Advancement of Military Medicine Inc, Bethesda, Maryland, USA; ^3^Center for Infectious Medicine, Department of Medicine Huddinge, Karolinska Institutet, Stockholm, Sweden

**Keywords:** ALVAC, MAIT cells, MVA, NK cells, poxvirus, viral vector

## Abstract

Recombinant poxviruses have been extensively studied as vaccine vectors, yet the specific mechanisms by which they engage the immune system remain incompletely understood. ALVAC is a poxviral vector that was a component of the HIV vaccine used in the Thai RV144 trial, showing modest efficacy in reducing HIV acquisition. Here, we show that in vitro ALVAC-HIV infection of peripheral blood mononuclear cells (PBMCs) activates natural killer (NK) and mucosal-associated invariant T (MAIT) cells. This activation was partially dependent on monocytes, cGAS sensing, and production of IL-18 and type I IFN. Furthermore, ALVAC-HIV-mediated activation of NK and MAIT cells contributed to the activation of B cells. Modified vaccinia Ankara (MVA), another poxviral vector used for prevention of smallpox and mpox, similarly activated NK and MAIT cells. Overall, this suggests a conserved mechanism by which NK and MAIT cells could contribute to the immunogenicity of poxviral vectors.

## 1. Introduction

The attenuated nonreplicating canarypox virus vector, ALVAC, was a key component of the HIV vaccine regimen used in the Thai RV144 trial, which administered ALVAC-HIV (vCP1521) and AIDSVAX B/E over 6 months. This Phase 3 trial showed 31% efficacy in preventing new HIV infections at 3 years postvaccination [1]. Subsequent studies showed that ALVAC-HIV improved both the cellular and humoral vaccine-induced immune responses [2, 3]. Modified vaccinia Ankara (MVA), is an attenuated replication incompetent poxvirus derived from vaccinia virus, and has been used in vaccines for the prevention of Ebola [4], smallpox, and mpox [5], and has been considered a candidate for several other infectious diseases [6]. The mechanisms by which these viral vectors stimulate innate immune responses to modulate adaptive immunity are incompletely understood.

The crosstalk between natural killer (NK) cells and adaptive immunity is complex and important for an effective immune response. NK cells can promote B-cell activation and immunoglobulin production through direct interaction or via cytokine secretion [7–10]. In mice, ALVAC-HIV-induced NK-cell production of IFN-γ increased vaccine-specific antibody levels [11]. Recently, mucosal-associated invariant T (MAIT) cells have been shown to provide B cell help [12, 13] and promote the maturation of dendritic cells [14, 15] via CD40L, as well as via the production of cytokines such as IL-21. Vaccination with the ChAdOx01 viral vector activated MAIT cells in an IL-18 and IFN-α-dependent manner, and in mice, MAIT activation was essential for induction of antigen-specific CD8 T cells [16]. Similarly, specific MAIT and NK-cell phenotypes have been associated with the adaptive immune response following mRNA vaccination in humans [17–19]. MAIT and NK-cell activation has also been reported 3 days postvaccination and associated with humoral immune responses [20]. Understanding the role of NK and MAIT cells to vaccine induced immunity, and understanding how their activation varies across vaccine platforms is critical for leveraging these populations to enhance immunogenicity.

In this study, we demonstrate that poxviral vectors activate human NK and MAIT cells in vitro in a manner dependent on monocytes, IL-18, type I IFN, and cGAS. Furthermore, NK and MAIT cell activation contributed to ALVAC-HIV-induced B-cell activation, suggesting that they may promote the induction of humoral immune responses by poxvirus-vectored vaccines.

## 2. Materials and Methods

### 2.1. In Vitro Infection

Human PBMCs were isolated from whole blood collected from healthy volunteers under protocol RV229, which was approved by the Walter Reed Army Institute of Research (WRAIR) Institutional Review Board. All participants provided written informed consent prior to collection. Deidentified samples were processed to isolate peripheral blood mononuclear cells (PBMCs) by Ficoll (GE Healthcare) gradient. Cryopreserved PBMCs were thawed and resuspended at 5 x 10^6^ cells/ml in RPMI-1640 containing 2.05 mM L-Glutamine (HyClones) supplemented with 10% FBS (Sigma). 200 μl of cells (1 x 10^6^ cells) were plated in 96-well plates. Cells were then infected with ALVAC-1521, ALVAC-1540-GFP, (both kind gifts from Sanjay Phogat, Sanofi Pasteur), or MVA-GFP (a kind gift from Patricia Earl and Bernard Moss from the Laboratory of Viral Diseases, National Institute of Allergy and Infectious Diseases [NIAID]) at an MOI of 5 for 18 h at 37°C and 5% CO_2_. For blocking assays, cells were treated with 10 mg/ml of anti-IL-12p70 (clone 24910, R&D systems), 1 mg/ml of anti-IL-18 (clone 125–2 H, MBL Life Science), or were pretreated for 2 h with the cGAS inhibitor G140 (InvivoGen) at 26 mM, or with 5 mg/ml of antiIFNAR2 (clone MMHAR-2, Invitrogen).

### 2.2. Flow Cytometry

Cells were centrifuged and surface stained for 15 min at room temperature with a combination of the following antibodies: anti-CD3 Alexa Fluor 700 (clone UCHT1, BD Bioscience), anti-CD3 PE Texas red (clone 7D6, Invitrogen), anti-CD4 APC-H7 (clone SK 3, BD Bioscience), anti-CD8 BV711 (clone RPA-T8, Biolegend), anti-CD14 BV510 (clone MΦP9, BD Bioscience), anti-CD16 BUV496 (clone 3G8, BD Bioscience), anti-CD19 BV510 (clone HIB19, Biolegend), anti-CD19 APC (clone HIB19, Fisher Scientific), anti-CD38 BUV737 (clone HB7, BD Bioscience), anti-CD56 PE-Cy7 (clone B159, BD Bioscience), anti-CD69 BUV395 (clone FN50, BD Bioscience), anti-CD161 BV421 (clone HP-3G10, Biolegend), anti-Vα7.2 BV785 (clone 3C10, Biolegend), and Aqua live/dead (Invitrogen). Cells were then washed and fixed with 1% formaldehyde solution. Flow cytometry data was collected on an LSRII flow cytometer (BD) and analyzed using FlowJo version 10.10.0 software for Mac OS (Becton Dickinson, Franklin Lakes, NJ, USA). Uniform Manifold Approximation and Projection (UMAP) was performed in FlowJo using concatenated data from 5 donors stimulated or not with ALVAC-HIV with an equal number of cells from each participant and condition.

### 2.3. Monocyte Isolation and Depletion

Monocytes were isolated and depleted from PBMCs using a human pan monocyte isolation kit (Miltenyi) following the manufacturer's instructions. Cells in the positive fraction containing monocyte-depleted PBMCs or in the negative fraction containing monocytes were counted and used for in vitro ALVAC infections.

### 2.4. NK and MAIT Cell Depletion

PBMCs were stained as previously described, and cells were run on a FACSMelody (BD). For NK-cell depletion, PBMCs were gated first on Aqua live/dead negative cells, and CD3-CD56- as well as CD3+ cells were sorted. For MAIT cell depletion, PBMCs were gated first on Aqua live/dead negative cells, and Vα7.2- as well as Vα7.2+CD161- cells were sorted. Cells were counted and used for in vitro ALVAC infections.

### 2.5. Soluble Markers Mesurements

Biomarkers were measured from culture supernatants using commercially available kits. Bio-Plex Pro Human Inflammation Panel 1 Luminex kits (BioRad, Hercules, CA, USA) were used to measure interferon-alpha 2 (IFN-α2), IL-29/interferon-lambda 1 (IFN-λ1), interferon beta (IFNβ), and IL-28a/interferon-lambda 2 (IFN-λ2).

### 2.6. Statistical Analysis

Statistical analysis was performed using GraphPad Prism version 10.1.1 for Mac OS. Comparisons between groups were performed using the Wilcoxon test, or the Friedman test when comparing more than two groups. *p* Values below 0.05 were considered significant.

## 3. Results and Discussion

**Figure 1:**
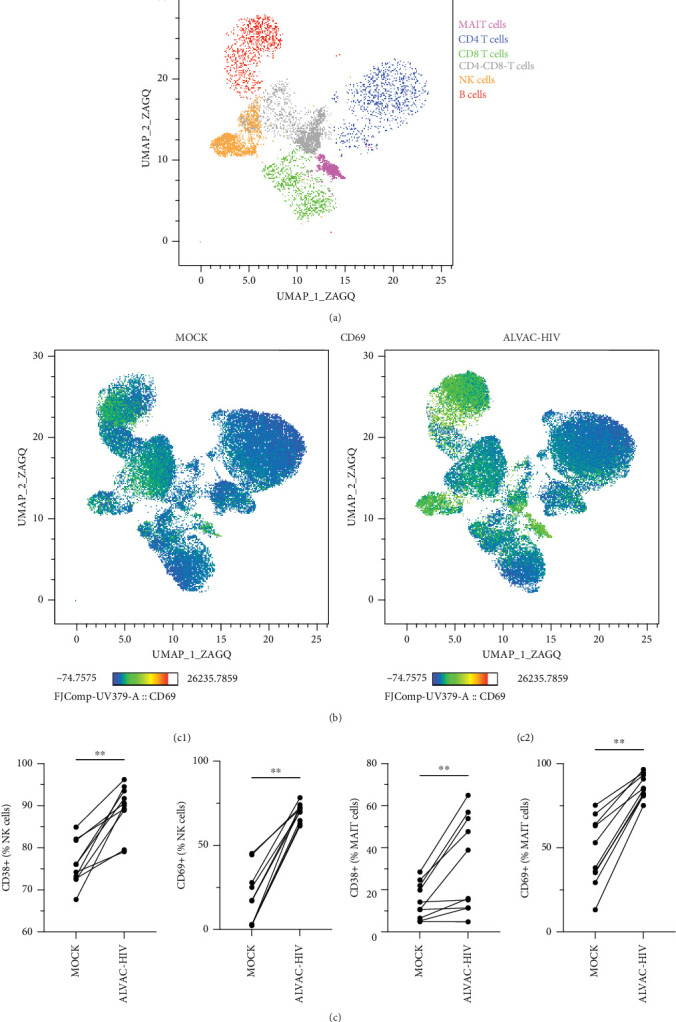
ALVAC-HIV infection of PBMCs activates NK and MAIT cells. (A) UMAP showing clusters corresponding to MAIT cells, CD4 T cells, CD8 T cells, CD4-CD8- T cells, NK cells, and B-cells generated from concatenated data from PBMCs of five individuals stimulated or not with ALVAC-HIV. (B) Heap map showing CD69 levels for MOCK or ALVAC-HIV treated PBMCs over the UMAP clusters. (C) Levels of CD38 and CD69 expression by NK cells (c1) and MAIT cells (c2) after ALVAC infection of PBMCs (*n* = 10). *⁣*^*∗∗*^*p*  < 0.01.

**Figure 2:**
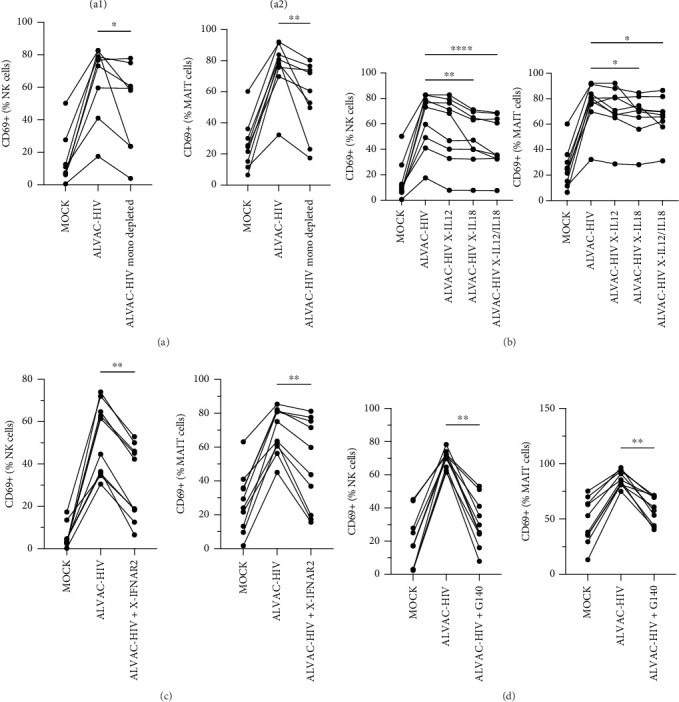
ALVAC-mediated NK and MAIT cell activation involves monocytes, IL-18, type I interferon, and cGAS. (A) Levels of CD69 expression by NK (a1) and MAIT (a2) cells after ALVAC-HIV infection of PBMCs or monocyte-depleted PBMCs (*n* = 8 for NK cells and *n* = 9 for MAIT cells), (B) levels of CD69 expression by NK and MAIT cells after ALVAC-HIV infection of PBMCs in the presence or absence of IL-12 and/or IL-18 blocking antibodies (*n* = 9), (C) IFNAR2 blocking antibody (*n* = 10), and (D) cGAS inhibitor G140 (*n* = 10). *⁣*^*∗*^*p*  < 0.05, *⁣*^*∗∗*^*p*  < 0.01, and *⁣*^*∗∗∗∗*^*p* < 0.0001.

To assess whether ALVAC may induce NK and MAIT cell activation, we stimulated human PBMCs with ALVAC-HIV and evaluated expression of the CD69 activation marker by flow cytometry. We used UMAP to visualize CD69 levels on the major lymphocyte populations. ALVAC-HIV mostly induced CD69 expression on NK cells, B-cells, MAIT cells, and CD4-CD8- T cells with limited induction on CD4+or CD8+T cells (Figure 1A,B). We then used manual gating to confirm ALVAC-HIV-induced upregulation of CD69, as well as CD38 as an additional activation marker, on the surface of both NK and MAIT cells (Figure 1C and Figure S1). Because CD69 was more strongly upregulated, we focused our analysis on this activation marker. ALVAC has a preferential tropism for monocytes [21]. Therefore, we subsequently investigated if depletion of monocytes impacts ALVAC-HIV-induced activation of NK and MAIT cells. Activation of both NK and MAIT cells was partially reduced in monocyte-depleted PBMC (Figure 2A). Because cytokines, such as IL-12 and IL-18 are capable of activating NK [22, 23] and MAIT [23–25] cells, we treated PBMCs with ALVAC-HIV in the presence of IL-12 and IL-18 blocking antibodies alone or in combination. Blocking IL-18 partially reduced NK and MAIT cell activation, while blocking IL-12 had no measurable effect (Figure 2B). Blocking both IL-12 and IL-18 had a similar effect as blocking IL-18 alone.

ALVAC-HIV has been reported to stimulate production of type I interferons [26], which are potent inducers of MAIT cell activation [25, 27]. Thus, we performed an experiment blocking the IFN-α/β receptor chain 2 (IFNAR2), to block type I interferon signaling. Similarly to IL-18, blocking the type I interferon receptor partially reduced NK and MAIT cell activation (Figure 2C). Because our results indicate a role for monocytes in activating NK and MAIT cells, we purified monocytes and treated them with ALVAC-HIV before measuring the levels of IFN-α2, IFNβ, IFN-λ1, and IFN-λ2 in the culture supernatant. Levels of IFNs were mostly below the detection limit in MOCK-treated cells and strongly increased when the monocytes were treated with ALVAC-HIV (Figure S2).

Previous work demonstrated that cGAS, a cytosolic DNA sensor, is involved in ALVAC sensing and induction of the type I IFN response [26]. Accordingly, we found that pretreating PBMCs with a cGAS inhibitor before infecting with ALVAC-GFP significantly reduced NK and MAIT cell activation (Figure 2D). This was not due to reduced infectivity of ALVAC-GFP, as shown by the lack of change in GFP expression (Figure S3).

Our results suggest that ALVAC-HIV-induced NK and MAIT cell activation are mediated in part by monocytes, IL-18, and type I interferon. This aligns with previous studies where adenovirus viral vectors induced MAIT [16] and γδ T [28] cell activation, and suggests a common innate immune activation pathway shared by different viral vectors. To further investigate this point and determine if other poxvirus viral vectors might also activate NK and MAIT cells, we infected PBMCs with MVA-GFP in vitro. Similar to the results obtained with ALVAC-HIV, NK, and MAIT cells showed an increased expression of CD69 after infection of PBMCs with MVA, and this response was reduced when PBMCs were pretreated with a cGAS inhibitor or a blocking antibody against IFNAR2 (Figure S4).

**Figure 3:**
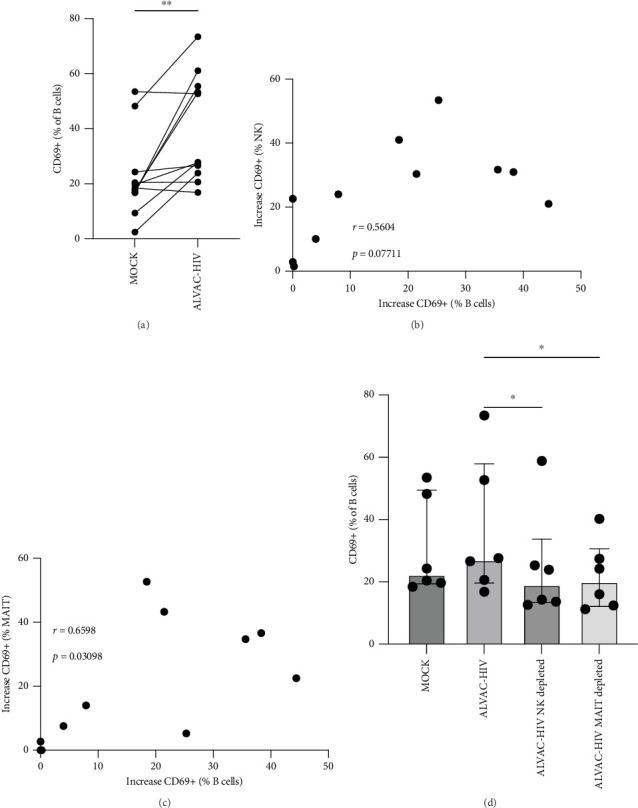
NK and MAIT cells contribute to ALVAC-HIV mediated B-cell activation. (A) Levels of CD69 expression by B-cells after 18 h of MOCK or ALVAC-HIV infection of PBMCs at a MOI of 5 (*n* = 11). Associations between the increase in CD69 expression by NK cells (B) or MAIT cells (C) and the increase in CD69 expression by B-cells (*n* = 11). (D) Levels of CD69 expression by B cells after 18 h of MOCK or ALVAC-HIV infection of PBMCs, NK-cell depleted PBMCs, or MAIT cell depleted PBMCs (*n* = 6). *⁣*^*∗*^*p*  < 0.05 and *⁣*^*∗∗*^*p*  < 0.01.

Next, we confirmed our initial observation that B cells displayed higher levels of CD69 after in vitro stimulation with ALVAC-HIV (Figure 3A and Figure S5). We therefore, investigated if there was a role for NK and MAIT cells in promoting B-cell activation following ALVAC-HIV stimulation. First, we found that the increase in activation of NK (*r* = 0.56; *p*=0.08; Figure 3B) and MAIT (*r* = 0.67; *p*=0.03; Figure 3C) cell was associated with the increase in B cell activation. Finally, we depleted PBMCs of either NK or MAIT cells prior to infection with ALVAC-HIV. Both NK and MAIT cell depletion abolished the induction of CD69 in B cells (Figure 3D). This result suggests that NK and MAIT cells might be part of an innate response pathway that promotes B-cell activation in response to poxvirus.

### 3.1. Data Limitations and Perspectives

Our analysis did not include induction of cytokine production by NK and MAIT cells and is limited to CD38 and CD69, two widely used activation markers [25, 29–32]. While, our results indicate an involvement for monocytes in producing IFNs, it is possible that other cells, such as dendritic cells, are also involved. Our work confirms previous studies that showed that MAIT cells can be activated by viral vector sensing and subsequent cytokine production [16, 25, 27]. The in vivo impact of NK and MAIT cell activation following poxvirus vaccination in humans on adaptive immunity remains to be investigated. Of note, NK-cell activation has been reported 7 days following MVA vaccination in a phase I trial [22]. Additional work is needed to determine the mechanisms used by NK and MAIT cells to promote B-cell activation following poxvirus infection or vaccination.

## 4. Concluding Remarks

In this study, we report that NK and MAIT cells are activated by in vitro infection with poxviral vectors. This activation involves sensing of the viral vector by cGAS and is dependent on monocytes, IL-18, and type I IFN. Furthermore, both NK and MAIT cells contributed to ALVAC-HIV-mediated B-cell activation. Adenovirus vectors have been reported to use a similar IL-18 and IFN pathway to activate MAIT cells [16], suggesting the presence of a conserved mechanism by which MAIT and NK cells may contribute to the immunogenicity of viral vectors. Understanding the mechanisms by which ALVAC and other viral vectors enhance vaccine-induced immune responses could provide valuable insights towards the improvement of vaccine efficacy.

## Data Availability

The data that support the findings of this study are available from the corresponding author upon reasonable request.

## Supporting Information

Supporting Information Figure S1. (A) Gating strategy for NK and MAIT cells. (B) Representative flow plots of CD38 and CD69 expression by NK and MAIT cells after 18 h of MOCK or ALVAC-HIV infection of PBMCs at a MOI of 5. Figure S2. Monocytes were treated with ALVAC-HIV for 18 h and levels of IFN-α2, IFN-β, IFN-λ1, and IFN-λ2 were measured in the culture supernatant (*N* = 4). Figure S3. Representative flow plots of GFP expression in PBMCs 18 h after ALVAC-GFP infection at a MOI of 5 (A). Levels of GFP-positive cells after treatment with G140 or anti-IFNAR2 (B) (*N* = 5). Figure S4. Levels of CD69 expression by NK (left) and MAIT (right) cells after MVA infection of PBMCs (B) (*N* = 5). Figure S5. Gating strategy for B-cells and representative flow plots of CD69 expression 18 h after MOCK or ALVAC-HIV infection at a MOI of 5.
