# Supplementary material for: Poxvirus Vectors Activate Human NK and MAIT Cells in a Type I Interferon, IL-18, and Monocyte-Dependent Manner
Source: J Immunol Res. 2025 Jul 12;2025:1203141. doi: 10.1155/jimr/1203141 (PMC12276059; doi:10.1155/jimr/1203141)
Supplement: Supporting Information — Figure S1. (A) Gating strategy for NK and MAIT cells. (B) Representative flow plots of CD38 and CD69 expression by NK and MAIT cells after 18 h of MOCK or ALVAC-HIV infection of PBMCs at a MOI of 5. Figure S2. Monocytes were treated with ALVAC-HIV for 18 h and levels of IFN-α2, IFN-β, IFN-λ1, and IFN-λ2 were measured in the culture supernatant (N = 4). Figure S3. Representative flow plots of GFP expression in PBMCs 18 h after ALVAC-GFP infection at a MOI of 5 (A). Levels of GFP-positive cells after treatment with G140 or anti-IFNAR2 (B) (N = 5). Figure S4. Levels of CD69 expression by NK (left) and MAIT (right) cells after MVA infection of PBMCs (B) (N = 5). Figure S5. Gating strategy for B-cells and representative flow plots of CD69 expression 18 h after MOCK or ALVAC-HIV infection at a MOI of 5. [file 1203141.f1.pdf]

Supplementary Figures.

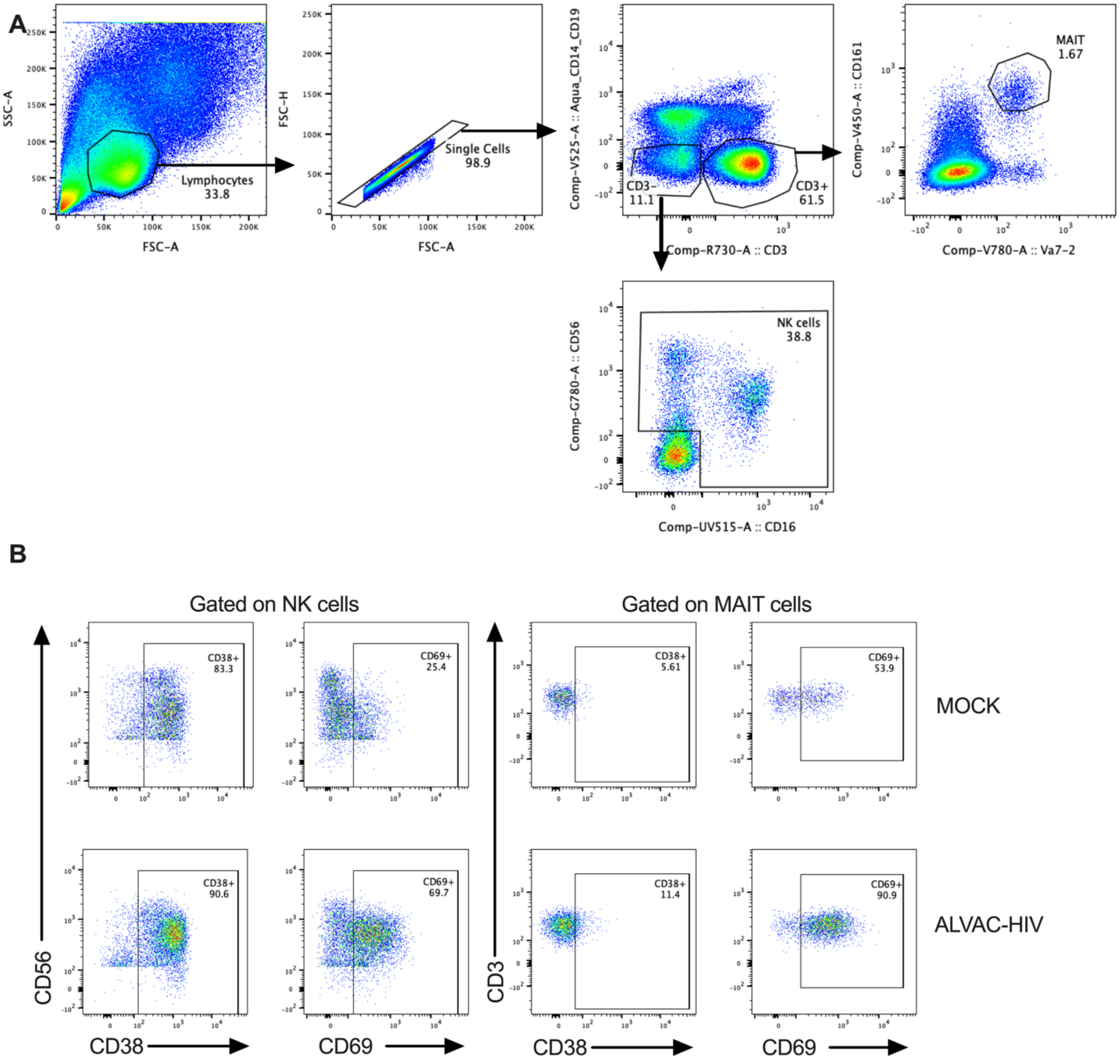

Supplementary Figure 1. (A) Gating strategy for NK and MAIT cells. (B)) Representative flow plots of CD38 and CD69 expression by NK and MAIT cells after 18 hours of MOCK or ALVAC-HIV infection of PBMCs at a MOI of 5.

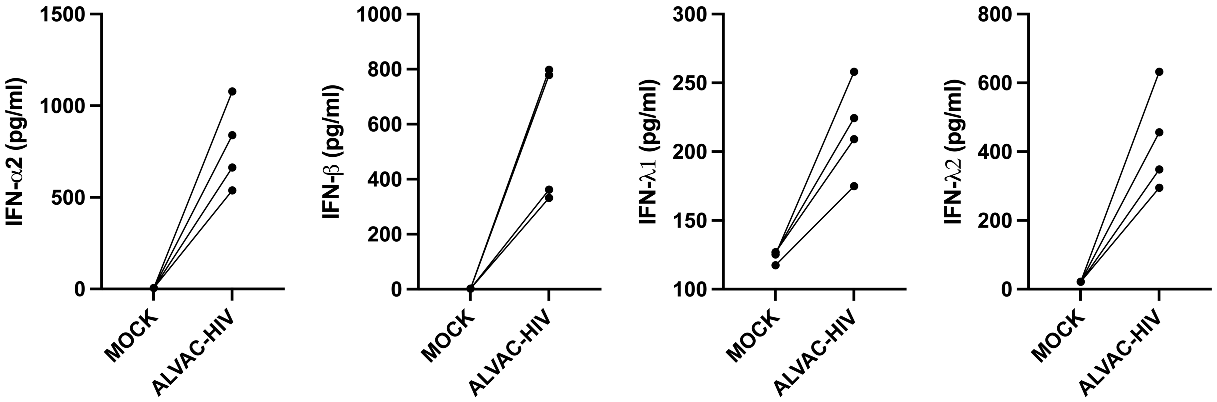

Supplementary Figure 2. Monocytes were treated with ALVAC-HIV for 18 hours and levels of IFN-α2, IFN-β, IFN-λ1, and IFN-λ2 were measured in the culture supernatant (N=4).

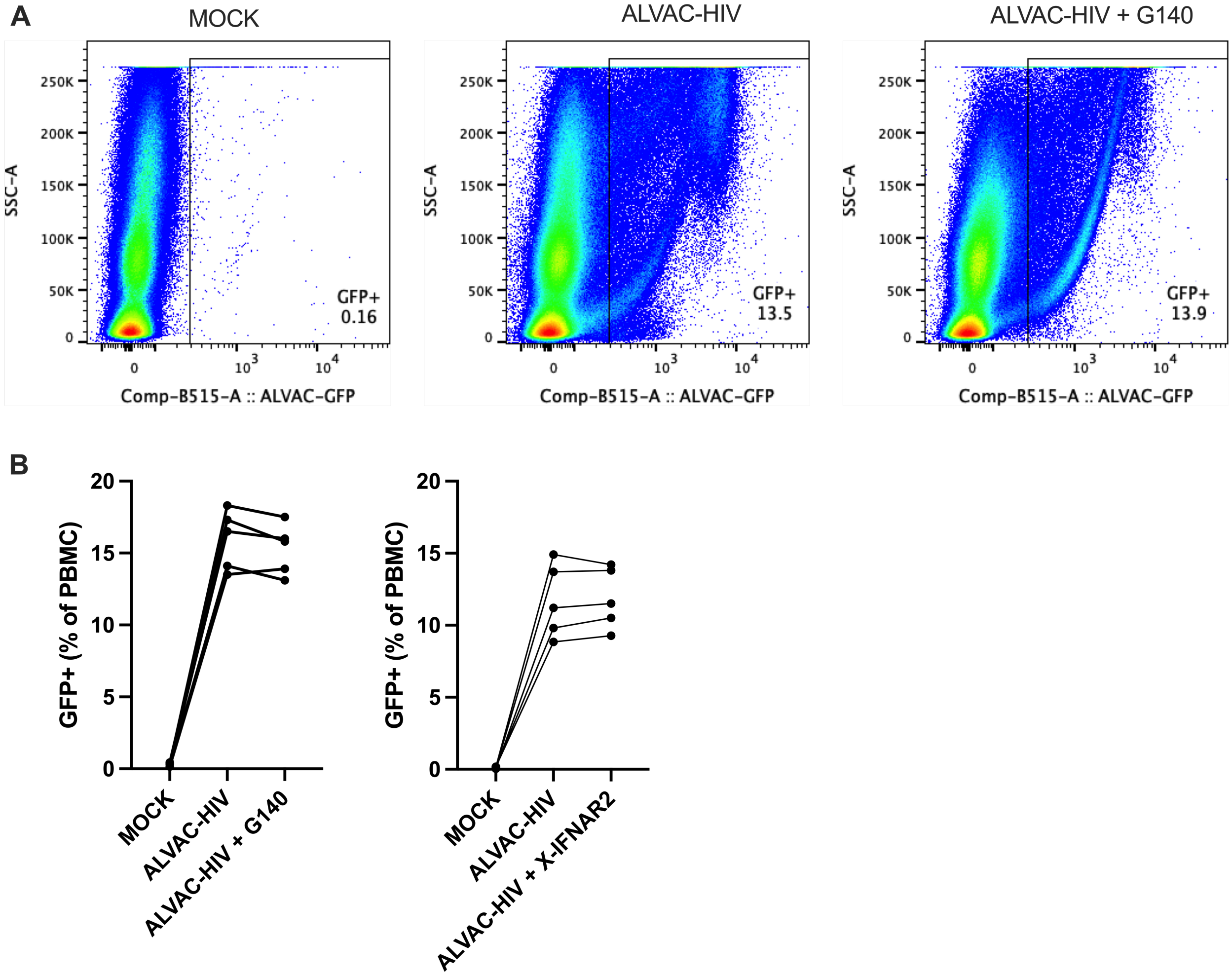

Supplementary Figure 3. Representative flow plots of GFP expression in PBMCs 18 hours after ALVAC-GFP infection at a MOI of 5 (A). Levels of GFP positive cells after treatment with G140 or anti-IFNAR2 (B) (N=5).

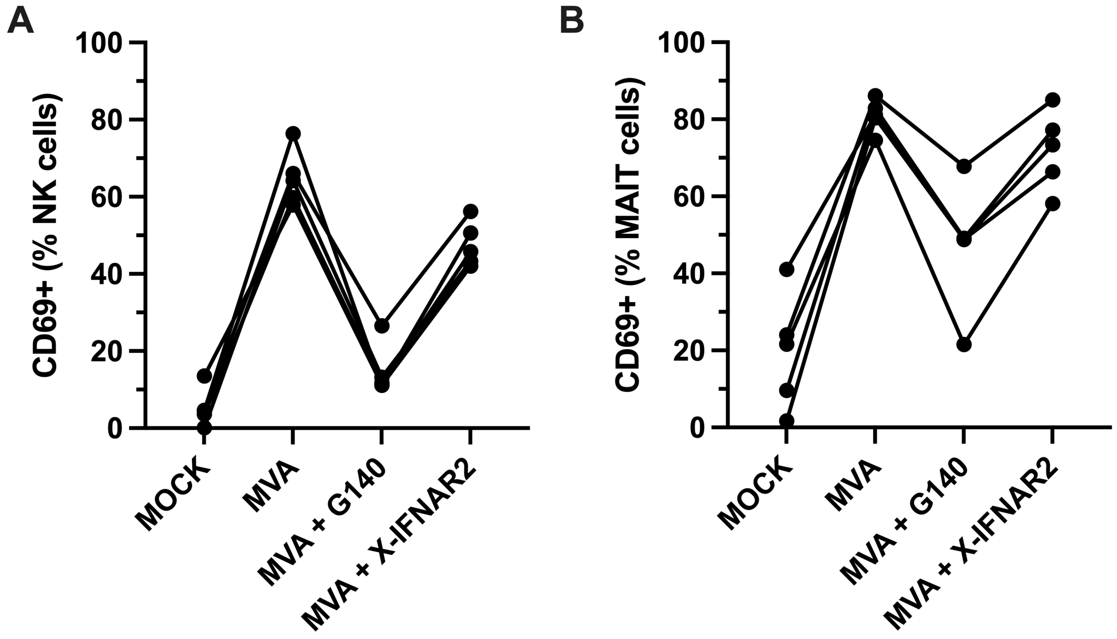

Supplementary Figure 4. Levels of CD69 expression by NK (left) and MAIT (right) cells after MVA infection of PBMCs (B) (N=5).

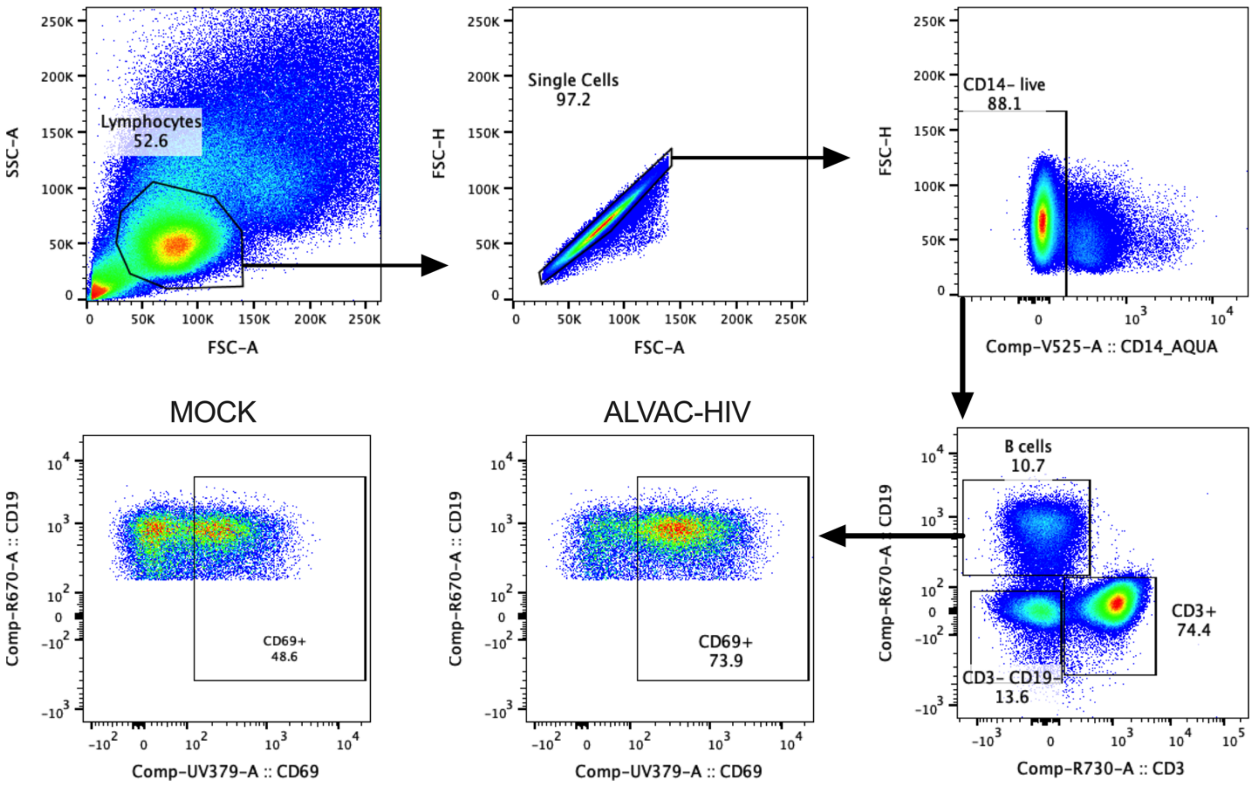

Supplementary Figure 5. Gating strategy for B cells and representative flow plots of CD69 expression 18 hours after MOCK or ALVAC-HIV infection at a MOI of 5.
